# Nosocomial outbreak by NDM-1-producing *Klebsiella pneumoniae* highly resistant to cefiderocol, Florence, Italy, August 2021 to June 2022

**DOI:** 10.2807/1560-7917.ES.2022.27.43.2200795

**Published:** 2022-10-27

**Authors:** Marco Coppi, Alberto Antonelli, Claudia Niccolai, Andrea Bartolini, Laura Bartolini, Maddalena Grazzini, Elisabetta Mantengoli, Alberto Farese, Filippo Pieralli, Maria Teresa Mechi, Vincenzo Di Pilato, Tommaso Giani, Gian Maria Rossolini

**Affiliations:** 1 Department of Experimental and Clinical Medicine, University of Florence, Florence, Italy; 2 Microbiology and Virology Unit, Florence Careggi University Hospital, Florence, Italy; 3 Hospital Infection Prevention and Control Unit, Florence Careggi University Hospital, Florence, Italy; 4 Infectious and Tropical Diseases Unit, Florence Careggi University Hospital, Florence, Italy; 5 Subintensive Care Unit, Florence Careggi University Hospital, Florence, Italy; 6 Department of Surgical Sciences and Integrated Diagnostics (DISC), University of Genoa, Genoa, Italy

**Keywords:** FDC, CPE, *K. pneumoniae*, *Enterobacterales*, resistance mechanism, *cirA*, nosocomial infections, carbapenemase

## Abstract

A nosocomial outbreak by cefiderocol (FDC)-resistant NDM-1-producing *Klebsiella pneumoniae* (NDM-Kp) occurred in a large tertiary care hospital from August 2021–June 2022 in Florence, Italy, an area where NDM-Kp strains have become endemic. Retrospective analysis of NDM-Kp from cases observed in January 2021–June 2022 revealed that 21/52 were FDC-resistant. The outbreak was mostly sustained by clonal expansion of a mutant with inactivated *cirA* siderophore receptor gene, which exhibited high-level resistance to FDC (MIC ≥ 32 mg/L) and spread independently of FDC exposure.

Cefiderocol (FDC) is one of the few novel antimicrobial agents active against multidrug-resistant Gram-negative bacteria strains producing metallo-beta-lactamases (MBLs) [1-3]. Thus far, acquired FDC resistance has been occasionally reported in sporadic cases [4-6]. Here, we report a nosocomial outbreak involving 21 patients caused by NDM-1-producing *Klebsiella pneumoniae* (NDM-Kp) resistant to FDC, which occurred in a large tertiary care Italian hospital from August 2021 to June 2022. We describe the features of the strains involved in this outbreak.

## Outbreak detection and investigation

In late 2018, an outbreak of NDM-producing Enterobacterales emerged in Tuscany in central Italy, involving several hospitals especially from the north-western area [7,8]. The outbreak was mostly sustained by strains of an ST147 NDM-Kp sublineage (named ST147-vir clone) which uniformly exhibited FDC susceptibility [9]. Since 2019, at our facility, the largest regional tertiary care hospital located in the central area of Tuscany, cases with NDM-Kp related to the regional outbreak have been recurrently observed following the transfer of infected or colonised patients from other hospitals or long-term-care facilities, with limited intra-hospital transmission.

Since its introduction in the hospital formulary (list of pharmaceutical agents and related information) in June 2021, FDC has been occasionally used for treating infections caused by multidrug-resistant Gram-negative pathogens, mostly based on presumed susceptibility. In vitro susceptibility to FDC was not routinely tested because of the accuracy issues reported with available commercial systems [10,11] and by the European Committee on Antimicrobial Susceptibility Testing (EUCAST) [12].

**Figure 1:**
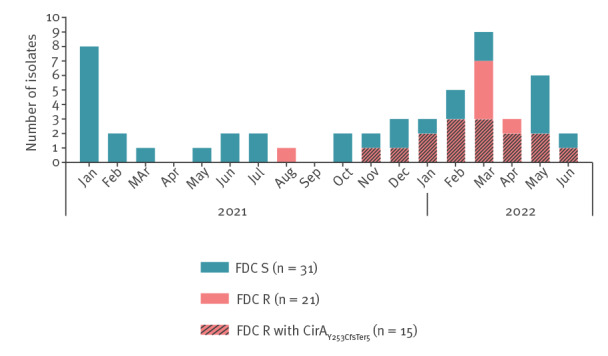
Number of retrospectively analysed non-replicate NDM-1-producing *Klebsiella pneumoniae* isolates by month of detection from a tertiary care hospital outbreak in Florence, Italy, January 2021–June 2022 (n = 52 isolates)

In June 2022, following the detection of an FDC-resistant NDM-Kp isolate (tested upon specific request of an infectious disease consultant), FDC susceptibility was retrospectively evaluated by reference broth microdilution (BMD) with iron-depleted medium [13] on NDM-Kp isolates from 52 cases observed since January 2021. This analysis revealed the emergence of FDC-resistant NDM-Kp in the second half of 2021, with a remarkable increase in the first months of 2022, i.e. a peak in January to March, during which 12 of 21 total FDC-resistant NDM-Kp isolates were observed (Figure 1; see Supplementary Table S1 for an overview of the clinical data of the cases). Among the resistant isolates, FDC minimum inhibitory concentrations (MICs) ranged from 4 to > 64 mg/L, with a bimodal distribution (see Supplementary Figure S1 for an overview of antimicrobial susceptibility test results).

FDC R: cefiderocol-resistant *Klebsiella pneumoniae* isolates; FDC R CirA_Y253CfsTer5_: cefiderocol-resistant, CirA_Y253CfsTer5_-positive *Klebsiella pneumoniae* isolates; FDC S: cefiderocol-susceptible *Klebsiella pneumoniae* isolates.

**Figure 2:**
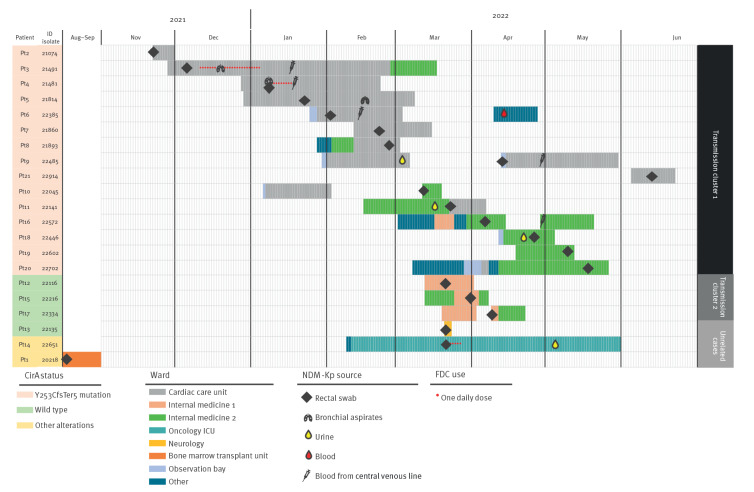
Timeline of cefiderocol-resistant NDM-1-producing *Klebsiella pneumoniae*-positive cultures during a tertiary care hospital outbreak in Florence, Italy, August 2021–June 2022 (n = 21 patients)

FDC: cefiderocol; ICU: intensive care unit; NDM-Kp: NDM-1-producing *Klebsiella pneumoniae*.

Of the 21 patients (11 men, 12 women; median age: 67 years (range: 45–91)) from which FDC-resistant NDM-Kp were isolated, all experienced colonisation and nine experienced infections including bloodstream infections (BSI; n = 5, all related to the central venous catheter) and/or urinary tract infections (UTI; n = 4) and/or lower respiratory tract infections (LRTI; n = 3) (Figure 2). Notably, none of the 21 cases had received FDC before isolation of FDC-resistant NDM-Kp.

CirA alterations, hospital wards, the NDM-Kp source specimen, and the eventual cefiderocol treatment were reported for each patient. Thirteen of 15 patients, positive for a CirA_Y253CfsTer5_ mutant, exhibited epidemiological links (overlapping period in the same ward) with the index case (Pt2) or among each other.

## Genomic analysis 

**Figure 3:**
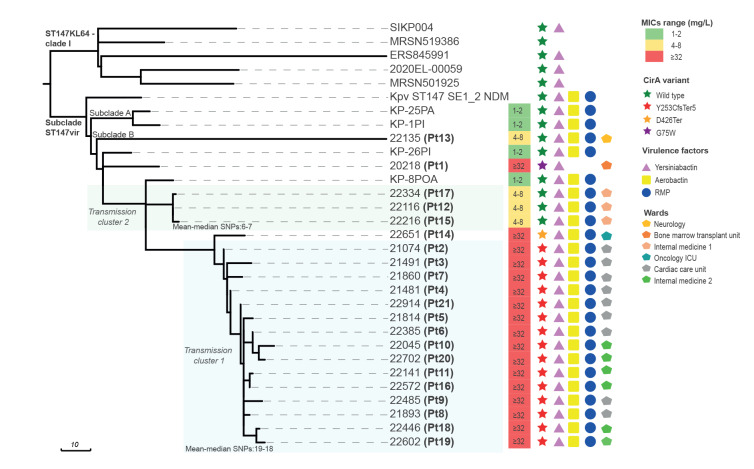
Phylogenetic analysis of cefiderocol-resistant NDM-1-producing ST147 *Klebsiella pneumoniae* from tertiary care hospital outbreak in Florence, Italy, August 2021–June 2022 (n=21)

ICU: intensive care unit; MIC: minimum inhibitory concentration; RMP: regulators of mucoid phenotype.

Whole genome sequencing (WGS) was carried out either on the first isolate from patients who experienced only carriage of FDC-resistant NDM-Kp, or on the first isolate from the most clinically relevant infection (BSI > LRTI > UTI), when more isolates were available from the same patient (n = 9 patients) (Figure 3). WGS was performed by Illumina Miseq (San Diego, United States) as previously described [9]. WGS analysis revealed that all the FDC-resistant NDM-Kp isolates belonged to ST147 and were related with the ST147-vir clone circulating in Tuscany [9] (Figure 3).

KP-1PI, KP-25PA, KP-26PI, KP-8POA were included as comparators belonging to the ST-147-vir clone circulating in Tuscany [9]. ERS845991 (from Hungary), MRSN519386 (from Middle East region), SIKP004 (from Thailand), MRSN501925 (from Germany), Kpv_ST147_SE1_2_NDM (from United Kingdom) and 2020EL-00059 (from United States) strains were included in the analysis as outliner representants of ST147 *K. pneumoniae.* The short-term bacterial evolution was generated using Gubbins v. 3.2.1 and RaxML tree builder. Branch lengths were reported in substitutions (point mutations) per genome. The complete genome of *K. pneumoniae* KP-1PI (Accession number CP071027), that was the first NDM-Kp ST147 isolated from a Tuscan region outbreak in 2018, was used as reference FDC susceptible strains for all in silico analyses [9].

Overall, 17 isolates carried alterations of the *cirA* siderophore receptor gene (Figure 3). Of these, 15 carried a 7-bp duplication (c.761_767dup) causing a frameshift followed by a premature stop codon (p.Y253CfsTer5), while singletons carried either a nucleotide deletion (c.1281del) leading to a premature stop codon (p.D426Ter) or a missense mutation (c.223G > T) leading to a G75W amino acid substitution (Figure 3; see Supplementary Figure S2 for the CirA amino acid sequence comparison). Interestingly, all the NDM-Kp with *cirA* alterations exhibited high-level FDC resistance (MICs: ≥ 32 mg/L) (Figure 3).

The remaining four isolates showing low-level FDC resistance (MICs: 4–8 mg/L) did not carry alterations in either *cirA* (Figure 3) or other genes for iron uptake systems (i. e. *fiu, fhuA, fepA, fbpA, efeO, exbB, exbD, fyuA, fitA, fur, iutA, iroN*), but carried missense mutations in the *baeS* (c.539C > T, p.T180I; n = 3) or in the *envZ* (c.109T > A; p.F37I; n = 1) genes encoding the sensor kinases of two component systems which regulate the expression of *cirA* and *fiu* iron-uptake genes [6,14]. Interestingly, missense mutations in these genes, although of different nature, were previously associated with FDC resistance [14].

Fine-tuned phylogenomics revealed the presence of two clusters of closely related isolates, likely originating through the clonal expansion of NDM-Kp with the CirA_Y253CfsTer5_ and with the BaeS_T180I_ alterations, respectively, while the singletons with other *cirA* alterations or with an altered *envZ* were more divergent (Figure 3).

The index CirA_Y253CfsTer5_ NDM-Kp (#21074_Pt2) clone was isolated in November 2021 from the surveillance swab of a patient transferred from another hospital in north-western Tuscany. Of the remaining 14 patients with CirA_Y253CfsTer5_ NDM-Kp isolates, 13 were observed in inpatients who were not colonised or infected by NDM-Kp at admission, while only one (#22045_Pt10) was found to be already positive at admission, in March 2022. Epidemiological links could be traced among the cases associated with CirA_Y253CfsTer5_ NDM-Kp, suggesting that this clone had entered the hospital with the index patient and had thereafter spread within wards attended by patients undergoing cardiac surgery. A colonised patient (#22045_Pt10), previously admitted for cardiac surgery, was the likely source for the subsequent dissemination of the CirA_Y253CfsTer5_ NDM-Kp clone in a medical ward, following readmission (Figure 2).

The index BaeS_T180I_ NDM-Kp strain (#22116_Pt12), isolated in March 2022 from the surveillance swab of a patient that was negative at admission, was associated with another cluster of transmission in another medical ward, while the three singletons were epidemiologically unrelated (Figure 2).

## Antimicrobial susceptibility testing

Antimicrobial susceptibility testing of isolates subjected to WGS, carried out by reference methods [15] and interpreted according to EUCAST clinical breakpoints [16], revealed that all FDC-resistant isolates were resistant to all tested beta-lactams and beta-lactam/beta-lactamase inhibitors combinations, aminoglycosides and fluoroquinolones, while 21/21 and 12/21 were susceptible to colistin and fosfomycin, respectively. The MICs of aztreonam/avibactam and tigecycline were 0.125–0.5 mg/L and ≤ 0.25–0.5 mg/L, respectively (i.e. in the susceptible range considering aztreonam clinical breakpoints and tigecycline pharmacokinetics/pharmacodynamics (PK/PD) breakpoints) (Table). Testing of FDC susceptibility by disk diffusion [17] could unambiguously categorise all FDC-resistant isolates (see Supplementary Table S1 for FDC disk diffusion test data).

**Table:** Minimum inhibitory concentrations (MIC) range, MIC_50_, MIC_90_ of cefiderocol-resistant NDM-producing *Klebsiella pneumoniae* isolates from a tertiary care hospital outbreak in Florence, Italy, August 2021–June 2022 (n = 21)

Antibiotics	AMC	AZA^a^	CAZ	CAA	CEP	CRO	CTA	ERT	MEM	PTZ	AMK	GNT	CIP	LEV	CLS	FOS	T/S	TIG^b^
MIC range (mg/L)	> 64	0.125 to 0.5	> 64	32 to > 64	> 16	> 4	> 64	2 to > 2	32 to > 64	64 to > 128	16 to > 16	> 8	> 1	> 8	≤ 0.5 to 1	4 to > 128	> 8/152	≤ 0.25 to 0.5
MIC_50_ (mg/L)	> 64	0.25	> 64	> 64	> 16	> 4	> 64	> 2	32	> 128	> 16	> 8	> 1	> 8	≤ 0.5	16	> 8/152	≤ 0.25
MIC_90_ (mg/L)	> 64	0.25	> 64	> 64	> 16	> 4	> 64	> 2	> 64	> 128	> 16	> 8	> 1	> 8	1	> 128	> 8/152	0.5
S	0	21	0	0	0	0	0	0	0	0	0	0	0	0	21	12	0	21

AMC: amoxicillin-clavulanic acid; AMK: amikacin; AZA: aztreonam-avibactam; CAA: ceftazidime/avibactam; CAZ: ceftazidime; CEP: cefepime; CIP: ciprofloxacin; CRO: ceftriaxone; CST: colistin; CTA: ceftolozane-tazobactam; ERT: ertapenem; FOS: fosfomycin; GNT: gentamicin; LEV: levofloxacin; MEM: meropenem; MIC: minimum inhibitory concentration; S: number of susceptible isolates; PTZ: piperacillin-tazobactam; T/S: trimethoprim-sulfamethoxazole; TGC: tigecycline.

^a^ Interpreted according to aztreonam clinical breakpoints [16].

^b^ Interpreted according to pharmacokinetics/pharmacodynamics (PK/PD) EUCAST clinical breakpoints [16].

MICs were obtained using reference broth microdilution or agar dilution for fosfomycin [15].

The resistance profile was consistent with the resistome of the isolates (see Supplementary Table S1 for the list of the acquired resistance genes revealed by the in silico analysis), which overall reflected that of the Tuscan ST147-vir clone [9], with minor inter-isolate variability.

The virulome profile of the FDC-resistant isolates (see Supplementary Table S1 for an overview of the virulence genes) was also consistent with that of the ST147-vir clone [9], except for the lack of the aerobactin siderophore genes (*iucABCD-iutA*) in the single isolate carrying the CirA_G75W_ alteration (Figure 3).

## Outbreak management

Following outbreak recognition in June 2022 and communication to the Regional and National Health Authorities, a bundle of interventions was implemented.

At the regional level (i) information about the outbreak was circulated among healthcare structures through the regional network for antimicrobial and diagnostic stewardship, (ii) FDC testing by disk diffusion [17] was recommended for all MBL-producing isolates of carbapenem-resistant Enterobacterales (MBL-CRE) from clinical specimens (and possibly also from surveillance specimens), followed by confirmation of results falling within the area of technical uncertainty (ATU) at reference regional laboratories and (iii) recommendations for treatment of infections by MBL-CRE were updated.

In addition, in our hospital (i) all MBL-CRE isolates categorised as FDC-resistant by disk diffusion or with a result falling within ATU were tested with reference BMD [13], (ii) enforcement of general infection prevention and control measures were emphasised and educational meetings were organised with healthcare personnel, (iii) all confirmed FDC-resistant isolates were subjected to genomic surveillance and (iv) a screening for the CirA_Y253CfsTer5_ mutant of NDM-Kp by real-time PCR was developed to rapidly analyse the resistant isolates (primers, probes, and reaction conditions are described in Supplementary Table S3 and Figure S3). 

During the subsequent period (1 July–15 October 2022), only two cases associated with an FDC-resistant NDM-Kp CirA mutants (one with Y253CfsTer5 and the other with D426Ter) were reported at our hospital, suggesting effectiveness of the intervention.

## Discussion

We report a hospital outbreak caused by FDC-resistant NDM-Kp observed in an area of Tuscany, Italy, where NDM-Kp is endemic. Since FDC is among the very few treatment options available for MBL-CRE infections, this is a worrying phenomenon in terms of resistance evolution.

By genomic surveillance, the outbreak was traced mostly to NDM-Kp strains highly resistant to FDC, carrying mutated *cirA* siderophore receptor genes with predominance of a CirA_Y253CfsTer5_ mutant, which was found in 15 of 21 cases, and with a minor contribution by mutants with other resistance mechanisms. All 21 FDC-resistant mutants were derivatives of the ST147 NDM-Kp sublineage involved in the large ongoing Tuscan outbreak, which emerged in late 2018 [7-9].

The FDC-resistant mutants had likely been selected outside our hospital. None of the 21 observed cases have been treated with FDC before isolation of the resistant NDM-Kp. Moreover, during this outbreak and in the preceding period (i.e. prior to introduction of FDC in the hospital formulary in June 2021), the use of FDC in our hospital remained very limited. Admission of patients already colonised by FDC-resistant NDM-Kp followed by intra-hospital transmission was the apparent source of this outbreak, underscoring the role that transfer of colonised or infected patients from other hospitals or long-term-care facilities could play in spreading of resistant pathogens, and the importance of active surveillance.

A recent study reported that a *cirA* mutant of NDM-Kp, selected in vitro, exhibited reduced fitness *vs* the parental strain in competition experiments, suggesting a low propensity to disseminate in the absence of sustained selective pressure [18]. However, our present findings indicate that at least some *cirA* mutants of NDM-Kp are able to spread in the hospital environment independently of a strong FDC selective pressure. Moreover, these strains can also retain a notable virulence potential, evident by the ability to cause nosocomial infections including BSI, LRTI and UTI. Further investigation regarding the fitness and virulence of the clone described in this study, as well as whether it carries compensatory mechanisms, may be necessary to explain this unexpected behaviour. One hypothesis could be that the lack of iron-catecholate outer membrane transporter CirA might be compensated by the presence of the structurally distinct siderophores yersiniabactin and aerobactin carried by the ST147-vir clone.

## Conclusions

The importance of surveillance, including genomic surveillance and active surveillance, and of strict compliance with infection prevention and control measures – based on a tight collaboration between clinical and laboratory personnel – are fundamental to avoid further spread of these difficult-to-treat Gram-negative bacteria and to preserve the activity of novel antimicrobials.

## Supplementary Data

509000 Supplementary Figures and Table S3

## Supplementary Data

39000 Supplementary Tables S1-2
