# Supplementary material for: Nosocomial outbreak by NDM-1-producing Klebsiella pneumoniae highly resistant to cefiderocol, Florence, Italy, August 2021 to June 2022
Source: Euro Surveill. 2022 Oct 27;27(43):2200795. doi: 10.2807/1560-7917.ES.2022.27.43.2200795 (PMC9615416; doi:10.2807/1560-7917.ES.2022.27.43.2200795)

## SUPPLEMENTARY APPENDIX 1

This supplementary material is hosted by *Eurosurveillance* as supporting information alongside the article **"Nosocomial outbreak by NDM carbapenemase-producing *Klebsiella pneumoniae* clone with inactivated *cirA* siderophore receptor gene, highly resistant to cefiderocol"**, on behalf of the authors, who remain responsible for the accuracy and appropriateness of the content. The same standards for ethics, copyright, attributions and permissions as for the article apply. Supplements are not edited by *Eurosurveillance* and the journal is not responsible for the maintenance of any links or email addresses provided therein.

Figure S1. *In vitro* activity of cefiderocol against NDM-producing *Klebsiella pneumoniae* clinical isolates, collected from Florence University Hospital in the period January 2021 (n=24) to June 2022 (n=28). When available, replicate isolates from the same patient always exhibited uniform FDC MICs (within ± 1 dilution).

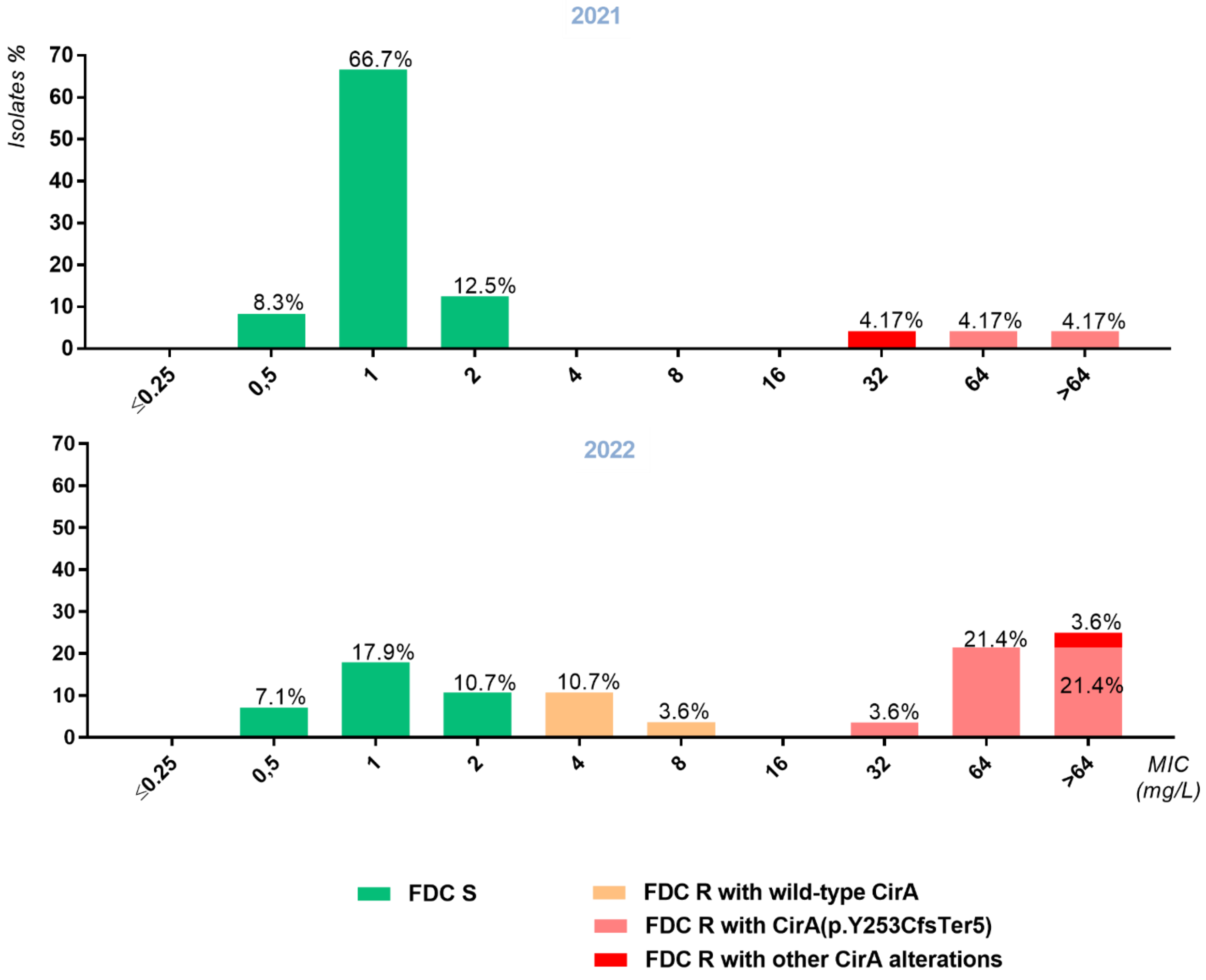

**Figure S2.** CirA aminoacidic sequences comparison of KP-1PI, as FDC susceptible reference strain, with the high-level FDC resistant isolates 20218 (p.G75W), 22651 (p.D426Ter) and 21074 (p.Y253CfsTer5), drew using *MView*.

```
                   cov    pid   1 [        .         .         .         .         .         .         .    *    . 80
CirA_WT_KP-1Pi 100.0% 100.0%      MFRLNPFIRAGLSASVVSLAFPALADVNEETLVVTASATEQNVKDAPASISVITQQDLQRKPVQNLKDVLRDVPGVQLTN
CirA_20218     100.0%  99.8%      MFRLNPFIRAGLSASVVSLAFPALADVNEETLVVTASATEQNVKDAPASISVITQQDLQRKPVQNLKDVLRDVPWVQLTN
CirA_22651      64.8% 100.0%      MFRLNPFIRAGLSASVVSLAFPALADVNEETLVVTASATEQNVKDAPASISVITQQDLQRKPVQNLKDVLRDVPGVQLTN
CirA_21074      39.1%  94.4%      MFRLNPFIRAGLSASVVSLAFPALADVNEETLVVTASATEQNVKDAPASISVITQQDLQRKPVQNLKDVLRDVPGVQLTN

                   cov    pid  81          .         .         .         .         .         .         :         . 160
CirA_WT_KP-1Pi 100.0% 100.0%      EGDNRKGVSIRGLSSSYTLILVDGKRVNSRNAVFRHNDFDLNWIPVDAIERIEVVRGPMSSLYGSDALGGVVNIITKKIG
CirA_20218     100.0%  99.8%      EGDNRKGVSIRGLSSSYTLILVDGKRVNSRNAVFRHNDFDLNWIPVDAIERIEVVRGPMSSLYGSDALGGVVNIITKKIG
CirA_22651      64.8% 100.0%      EGDNRKGVSIRGLSSSYTLILVDGKRVNSRNAVFRHNDFDLNWIPVDAIERIEVVRGPMSSLYGSDALGGVVNIITKKIG
CirA_21074      39.1%  94.4%      EGDNRKGVSIRGLSSSYTLILVDGKRVNSRNAVFRHNDFDLNWIPVDAIERIEVVRGPMSSLYGSDALGGVVNIITKKIG

                   cov    pid 161          .         .         .         .         .         .         .         . 240
CirA_WT_KP-1Pi 100.0% 100.0%      QKWTGTLSADTTIQEHRDRGDTYNGQFFTSGPLIDGVLGMKAYGSLAKRAKDDPQSSSNATGETPRIEGFTSRDGNVEFA
CirA_20218     100.0%  99.8%      QKWTGTLSADTTIQEHRDRGDTYNGQFFTSGPLIDGVLGMKAYGSLAKRAKDDPQSSSNATGETPRIEGFTSRDGNVEFA
CirA_22651      64.8% 100.0%      QKWTGTLSADTTIQEHRDRGDTYNGQFFTSGPLIDGVLGMKAYGSLAKRAKDDPQSSSNATGETPRIEGFTSRDGNVEFA
CirA_21074      39.1%  94.4%      QKWTGTLSADTTIQEHRDRGDTYNGQFFTSGPLIDGVLGMKAYGSLAKRAKDDPQSSSNATGETPRIEGFTSRDGNVEFA

                   cov    pid 241          .         .         .         .         .         .         .         . 320
CirA_WT_KP-1Pi 100.0% 100.0%      WTPNENHDFTAGYGFDRQDRDSDSLDRNRLERENYSLSHNGRWDIGNSELKFYGEKVDNKNPGQSGTITSESNAIDGKYV
CirA_20218     100.0%  99.8%      WTPNENHDFTAGYGFDRQDRDSDSLDRNRLERENYSLSHNGRWDIGNSELKFYGEKVDNKNPGQSGTITSESNAIDGKYV
CirA_22651      64.8% 100.0%      WTPNENHDFTAGYGFDRQDRDSDSLDRNRLERENYSLSHNGRWDIGNSELKFYGEKVDNKNPGQSGTITSESNAIDGKYV
CirA_21074      39.1%  94.4%      WTPNENHDFTAGC-------------RLRL--------------------------------------------------

                   cov    pid 321          .         .         .         .         .         .         .         . 400
CirA_WT_KP-1Pi 100.0% 100.0%      LPLGMINQLVTFGGEWRHDKLKDPVNLSSGGQSTSASQYALFIEDEWRIIEPLALTTGIRMDDHQTYGDHWSPRAYLVYN
CirA_20218     100.0%  99.8%      LPLGMINQLVTFGGEWRHDKLKDPVNLSSGGQSTSASQYALFIEDEWRIIEPLALTTGIRMDDHQTYGDHWSPRAYLVYN
CirA_22651      64.8% 100.0%      LPLGMINQLVTFGGEWRHDKLKDPVNLSSGGQSTSASQYALFIEDEWRIIEPLALTTGIRMDDHQTYGDHWSPRAYLVYN
CirA_21074      39.1%  94.4%      --------------------------------------------------------------------------------

                   cov    pid 401          .         .         .         .         :         .         .         . 480
CirA_WT_KP-1Pi 100.0% 100.0%      ATDTVTVKGGWATAFKAPSLLQLNPDWTTNSCRGSCSIVGNPDLKPETSESFELGLYYRGEEGWLENVEGSITTFQNNVD
CirA_20218     100.0%  99.8%      ATDTVTVKGGWATAFKAPSLLQLNPDWTTNSCRGSCSIVGNPDLKPETSESFELGLYYRGEEGWLENVEGSITTFQNNVD
CirA_22651      64.8% 100.0%      ATDTVTVKGGWATAFKAPSLLQLNPD------------------------------------------------------
CirA_21074      39.1%  94.4%      --------------------------------------------------------------------------------

                   cov    pid 481          .         .         .         .         .         .         :         . 560
CirA_WT_KP-1Pi 100.0% 100.0%      DMIDVLRTSSASEAPGYPNFVGWKTVNGKRVPIFRYFNVNKARIKGVETEVKIPFGDEWKLTVNYTYNDGRDLSNGGDKP
CirA_20218     100.0%  99.8%      DMIDVLRTSSASEAPGYPNFVGWKTVNGKRVPIFRYFNVNKARIKGVETEVKIPFGDEWKLTVNYTYNDGRDLSNGGDKP
CirA_22651      64.8% 100.0%      --------------------------------------------------------------------------------
CirA_21074      39.1%  94.4%      --------------------------------------------------------------------------------

                   cov    pid 561          .         .         .         .         .         .         .         . 640
CirA_WT_KP-1Pi 100.0% 100.0%      LQTLPFHTANGTLDWKPLDDWSFYVTANYTGQQRAVSATGKTPGGYTLFDVGAAWQVTKNVKLRSGVQNVGDKDLSRDDY
CirA_20218     100.0%  99.8%      LQTLPFHTANGTLDWKPLDDWSFYVTANYTGQQRAVSATGKTPGGYTLFDVGAAWQVTKNVKLRSGVQNVGDKDLSRDDY
CirA_22651      64.8% 100.0%      --------------------------------------------------------------------------------
CirA_21074      39.1%  94.4%      --------------------------------------------------------------------------------

                   cov    pid 641          .      ] 657
CirA_WT_KP-1Pi 100.0% 100.0%      SYTEEGRRYFMAVDYRF
CirA_20218     100.0%  99.8%      SYTEEGRRYFMAVDYRF
CirA_22651      64.8% 100.0%      -----------------
CirA_21074      39.1%  94.4%      -----------------
```

**Table S3.** Primers, probes and reaction conditions for the molecular detection of the $CirA_{Y253CfsTer5}$ mutants.

| *Oligonucleotide name* | *Sequence (5′-3′)* | *Final concentration in reaction mix (nM)* |
|---|---|---|
| *cirA-rt-fwd* | AAGGACGATCCGCAGTCATC | 500 |
| *cirA-rt-rev* | GGCTCAGAGAGTAGTTCTCC | 500 |
| *cirA-AOUC-rt-p* | FAM-GCAGGCTGCAGGCTACGG-MGBNFQ | 100 |

The reaction volume is 16 µL, including 3 µL of DNA (obtained with thermal lysis of a water bacterial suspension) approaches. The amplification program consisted of 35 two-step cycles of 15s at 95°C and 60s at 66 °C.

**Figure S3**. PCR amplification curve for the molecular detection of the $CirA_{Y253CfsTer5}$ mutants. A sample positive for the c.761_767 duplication in *cirA* gene is characterized by a higher intensity of fluorescent emission than samples with the wild type *cirA*, which are revealed with lower efficiency by probes.

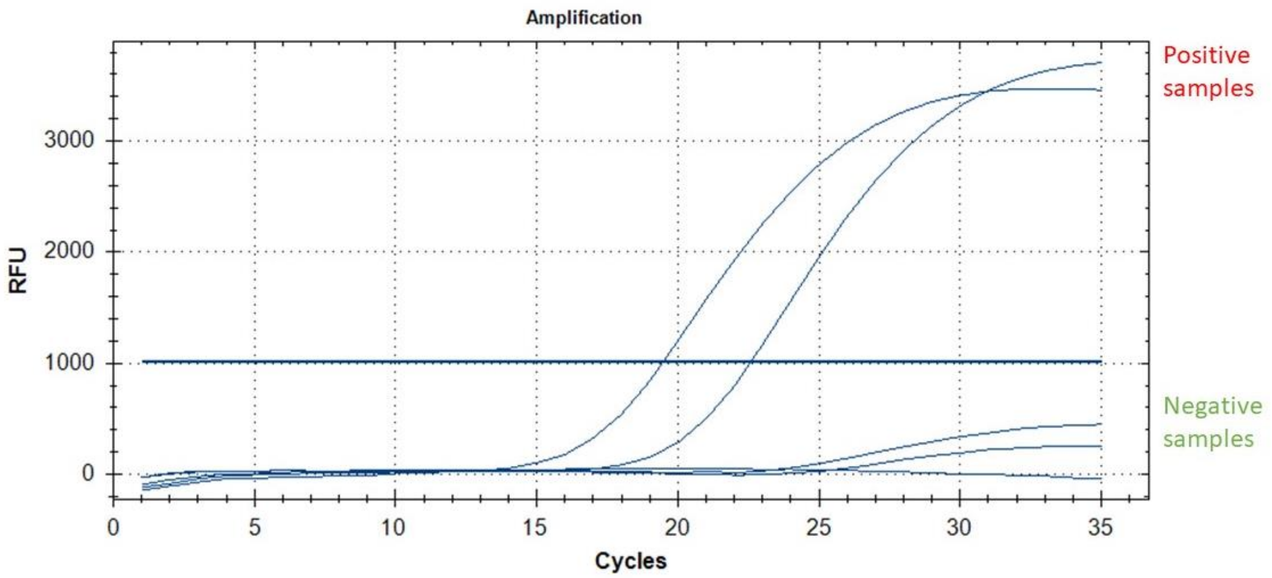

Supplement: Supplementary Figures and Table S3 [file 22-00795_ROSSOLINI_Supplementary_Figures_S1-3_and_Table_S3.pdf]
